# Asymmetric effects of social and economic incentives on cooperation in real effort based public goods games

**DOI:** 10.1371/journal.pone.0249217

**Published:** 2021-04-14

**Authors:** Jakob Hackel, Hitoshi Yamamoto, Isamu Okada, Akira Goto, Alfred Taudes

**Affiliations:** 1 Research Institute for Cryptoeconomics, University of Economics and Business, Vienna, Austria; 2 Faculty of Business Administration, Rissho University, Tokyo, Japan; 3 Faculty of Business Administration, Soka University, Tokyo, Japan; 4 School of Information and Communication, Meiji University, Tokyo, Japan; Kyushu Daigaku, JAPAN

## Abstract

Many practitioners as well as researchers explore promoting environmentally conscious behavior in the context of public goods systems. Numerous experimental studies revealed various types of incentives to increase cooperation on public goods. There is ample evidence that monetary and non-monetary incentives, such as donations, have a positive effect on cooperation in public goods games that exceeds fully rational and optimal economic decision making. Despite an accumulation of these studies, in the typical setting of these experiments participants decide on an allocation of resources to a public pool, but they never exert actual effort. However, in reality, we often observe that players’ real effort is required in these public goods game situations. Therefore, more analysis is needed to draw conclusions for a wider set of incentive possibilities in situations similar to yet deviating from resource allocation games. Here we construct a real effort public goods game in an online experiment and statistically analyze the effect different types of incentives have on cooperation. In our experiment, we examine combinations of monetary and social incentives in a setting aimed closer to practical realities, such as financial costs and real effort forming part of the decision to cooperate on a public good. In our real effort public goods game participants cooperate and defect on image-scoring tasks. We find that in our setting economic and social incentives produce an asymmetric effect. Interestingly economic incentives decreased the share of highly uncooperative participants, while social incentives raised the share of highly cooperative participants.

## Introduction

Researchers and practitioners alike have been working for decades on ever expanding frameworks that can help public goods to thrive by fostering collaboration. Through rising environmental concerns and evolving digitization new pathways to achieve better sustained systems are explored. One such pathway lies in experimental tests of the effects that different incentive models can have on the structure and sustenance of a population sharing a public good. Despite this focus and effort there is still much to be explored and learned. While many studies and experiments have considered typical settings of public goods, where participants allocate pre-allocated resources to a public pool, we find little accumulation of studies where real effort is incorporated into the costs and structure of such settings. It is our belief that there is ample knowledge to be learned from such a-typical settings. Even though standardization and comparability is yet not fully agreed on, we see future studies expanding these settings to further cover various factors incorporating real world considerations.

This study evolved out of a cooperation on a project with the City of Vienna to investigate an incentive based mobility system. In this system, the City of Vienna honors distance traveled when using sustainable transport modes with a non-monetary token. This token can be used to access cultural offers provided by theaters and museums in Vienna. To succeed with such a token various incentive models have to be considered. Further research on public goods variations could contribute to clarify incentive considerations to help promote contribution in such systems. We consider environmentally conscious behavior in the framework of cooperation in public goods games and thus explore incentives and settings in the economic games literature.

We are able to draw on extensive literature on public goods games, including exhaustive review papers [1–3]. Incentives are an essential mechanism to promote cooperation in public goods games. Incentives can be positive, as rewards, or negative, as punishment [4–7]. Many studies have explored the differences in the effects of rewards and punishment on cooperation [8–13]. However, there are two hurdles that need to be overcome when applying these results to real societal issues. One is the limitation of practical difficulty of punishment in voluntary participation systems, the other is the necessity of institutional incentives. For example, participation in an incentive based mobility system is up to the individual, and it is not practical to directly punish those who do not participate. Therefore, it is necessary to focus on the effect of rewards as positive incentives. A previous study has pointed out the necessity of rewards in a web-based service which is based on voluntary participation [14]. In addition, previous experimental studies have dealt with peer sanctions among participants, as theoretical and experimental literature have revealed the effects of institutional incentives [15–19]. In the real society, it is unrealistic to design a system based on peer sanctioning, and it is necessary to focus on the effects of institutional incentives. Institutional positive incentives can be rephrased as extrinsic incentives. How extrinsic incentives work in prosocial behavior should be considered to implement a system in the real society [20]. Additionally, behaviour in lab-based environments compared to situations in the field was found to be mostly consistent in previous studies [17, 21]. However, some suggest that differences can accrue when measuring lab and field results [22].

In these economic games the outcome can depend on several factors. We argue, that despite these studies, effective incentives in public goods games do not necessarily correspond to those in a situation that incorporates real effort. Therefore it is necessary to examine settings closer to a real world environment when possible. We attempt to analyze such a setting incorporating real effort in a public goods task. On task based economic games we can find differences in dictator games depending on subjects earning their endowments via tasks, which reduces full defectors, compared to windfall endowments [23]. Yet there is also evidence that when subjects first earn their endowments via a quiz there might be no difference, similarly when endowments were earned via tasks they did not induce a change in behavior [23, 24]. In our study we explore subjects’ behavior when another abstraction is removed, as the effort spent is a more direct contribution towards the public good. Additionally, we vary incentive combinations with economic costs and social incentives, via donations to a charity.

Previous studies have examined various economic games with respect to the behavior of participants in situations facing differing incentivization. In dictator games donations provided as a type of incentive produced a positive effect on contributions, while in a principal-agent situation workers reciprocated with effort to match donations in a CSR (corporate social responsibility) context [25, 26]. An increase in productivity was shown in a real effort experiment when donations were provided as incentives [27]. Most recently, two variations of donation incentives were examined in public goods games, with significantly higher contributions when donations were financed by the experimenter as well as an increase in productivity when financed by the subjects themselves [28]. Additionally, a difference in contributions when comparing donations to social versus non-social projects was shown, further implying a clear trend in positive effects for social incentives.

Our paper aims to further the discussion on the provision of incentives to skew the distribution of cooperation versus free-riding in public goods games. We conduct a real effort based online experiment in a setting similar to a public goods game and test two categories of incentives as well as their combinations. We choose a setting differing in several aspects from a traditional public goods game. In our setting too, participants receive an initial allowance. However, our participants do not contribute to the common pool by making an investment. Subjects contribute with their effort mainly. Rewards for completing a task successfully are shared among their group and depending on the treatment the subject themselves. Depending on the treatment the subjects may receive an additional social incentive, as a donation is made for each successfully solved task.

In addition to incentives, traditional psychological attitudes also encourage prosocial behavior. It is known that altruistic behavior is brought about by psychological attitudes such as reciprocity and generalized trust [29–31]. To analyze the effects of incentives in detail, we analyze the effects of two types of incentives on cooperative behavior after generalized reciprocity and generalized trust are controlled.

We consider this environment to be closer to a real life public good, as there is often both a financial investment and real effort needed to sustain public goods. Similarly to a traditional public goods game, the Nash equilibrium for subjects is to free-ride by not starting new tasks. The experiment is conducted on the Japanese Yahoo Crowdsourcing platform, where participants perform tasks in exchange for payment. Further details on the experimental setup can be found in the methods section.

Our results show that there are two distinct changes in behavior in this setting. First, introducing donations as additional incentives raises the share of full cooperators. Second, removing the resource cost reduces the share of full defectors. These effects may show a trend where economic incentives reduce defection, while social incentives promote cooperation.

## Methods

### Overview of the experiment

In our experiment, we construct four treatments for a real effort based public goods game. Contrary to a traditional setup, where participants contribute to a common pool from their individual budget, in our real effort setting participants contribute with their individual work effort to a public good. They receive varying incentive types to further identify trends among each treatment. Participants are split into fixed groups and contribute towards the benefit of the group. Participants are able to start and try to solve at most 100 tasks but are able to stop working and free ride at any point during the experiment. Participants are informed of the result and awarded payments after the experiment based on the work of their group members. Participants are not informed on their groups work in-between rounds.

In four treatments, we conduct all possible configurations of the cases with/without economic incentives and with/without social incentives. If a participant in any treatment solves a task correctly, the rest of his group will receive a reward. On top, depending on the treatment, a participant can have an additional individual incentive (being included in the group reward, or a donation to the Red Cross) depending on his treatment. Thus, we define participants to be treatment A if they are given neither economic nor social incentives, treatment B those with social incentives only, treatment C those with economic incentives only, and treatment D those who are given both economic and social incentives. Participants in treatment B or D (the social condition) are given additional individual incentives as a manager of this experiment will make a donation to the Red Cross depending on the number of correctly solved tasks, while participants in treatment A or C (the no social condition) are not given such an incentive. It should be noted that in the social condition, participants do not receive any tangible rewards from donating to the Red Cross. They are just given a chance to contribute to an altruistic reward.

The experiments were approved by the Research Ethics Committees at Rissho University (Approval number 02-01) and were performed in accordance with relevant guidelines and regulation. Participation required written informed consent. The experiment was conducted using oTree [32] and analyzed using HAD 16.02 [33].

### Experimental procedures

Participants were recruited through a Japanese crowdsourcing service (Yahoo! Crowdsourcing). The experiment started at 2pm (JPT) on April 15, 2020 and finished at 3:50pm the same day. Participants were able to find the survey under the title: “Economic games (with variable rewards based on points earned and surveys”. A total of 623 people participated in the experiment while 353 participants finished the experiments sucessfully. Among them, we adopt the data of those 242 participants that answered the demographics section and manipulation check completely for our statistical analysis. The average age of participants was 44.73 years (standard deviation: 11.31 years) and 59.7% were male. Participants on average earned JPY 23.06 (standard deviation: JPY 8.47).

**Fig 1:**
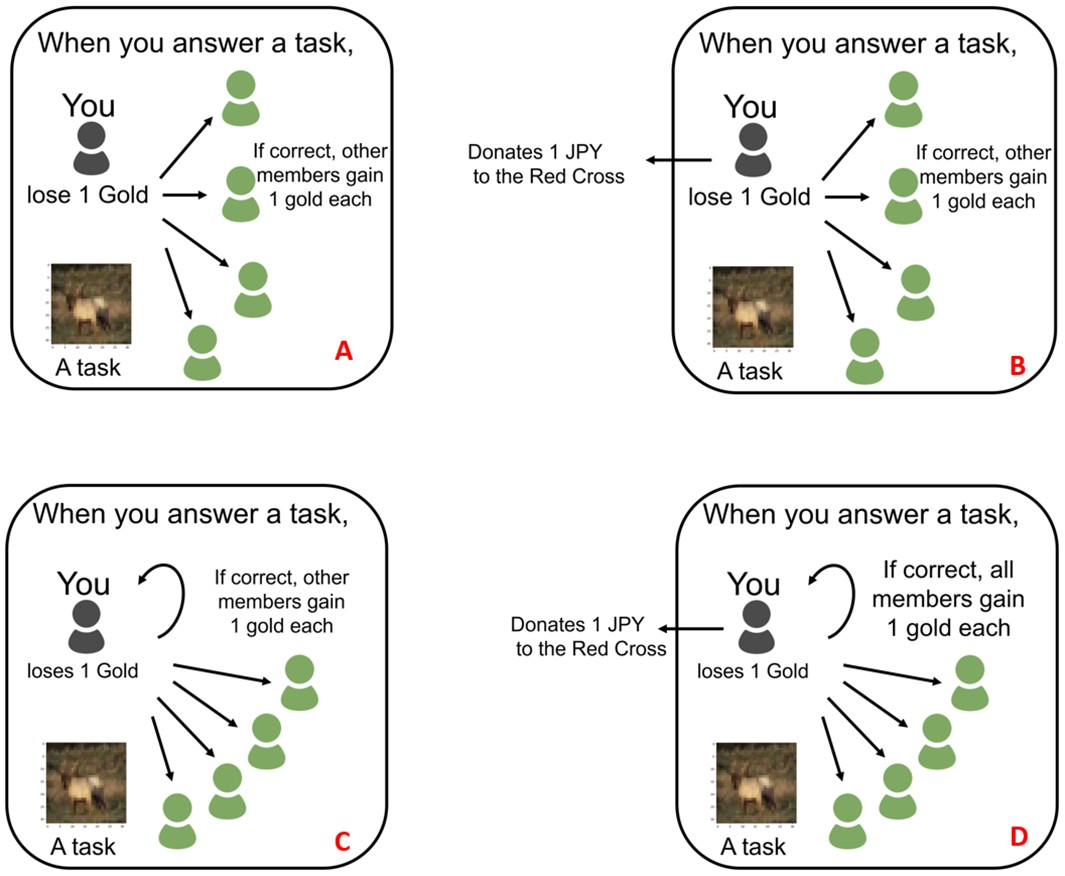
Punch images shown as instructions to each of the four treatments. Matrix showing the instructions to participants depending on their treatment. Starting from top left in clockwise rotation: Treatment A, B, C, D.

First, participants are each randomly assigned to one of four treatments. Additionally, each participant is randomly assigned to a party of five. They are shown each instruction screen in Japanese depending on their respective treatment. See S1 Text for an English translation where the text highlighted (bold style and colour in red) corresponds to the original version. After that screen, participants perform tasks on image recognition which is based on the CIFAR-10 dataset [34]. Each participant is able to start and finish a maximum of 100 tasks on image recognition, but is able to stop and defect at any time. Each participant receives information about the reward scheme that is applied to his effort. In the case of treatment A, the participant is shown punch images that explain how each task he solves results in rewards for only the rest of the group, while each task another group member solves results in rewards for the group except said group member. See Fig 1 for English versions of each reward scheme in a matrix.

Task sessions finished with a questionnaire including items about their demographic background shown in S1 Text.

After grouping each public goods game and calculating each payoff, each participant was provided with a link to receive their earned PayPay Bonus Light. A show-up fee of 2 PayPay Bonus Light was added to the earnings. 1 PayPay Bonus Light corresponds to 1 JPY and can be used for cashless payments, with a self-reported 35m users [35]. In addition, the participants of treatment B and D (the social condition) were informed of the donation made to the Red Cross by the manager (JPY 9,100 at May 16. Donation number is K014-00508792).

### Sampling frame

As with any online and lab experiment, there is a potential for sampling bias due to the population that is available. Online crowd-working experiment have seen a surge over the past decade and can bring great advantages. It is important to note however, that such online experiments are excluding to anyone without access to internet. In Japan around 80% of the population are considered to be using the internet [36]. Online experiments are also somewhat excluding to people with very limited technical abilities, which hinders access to such an online platform. It is usually older age groups which score lower on internet usage and technical ability surveys, which we consider to be a limitation of online experiments. While participants were paid in platform money, which can limit interested participants if restrictive, said e-money is used by over 35 million users and can be readily exchanged to Japanese Yen. We developed an experimental environment in which only the incentives differed, and subjects were assigned to each condition at random. We have also conducted an analysis controlling for psychological attitudes that may influence behaviour (Table 2).

## Results

**Fig 2:**
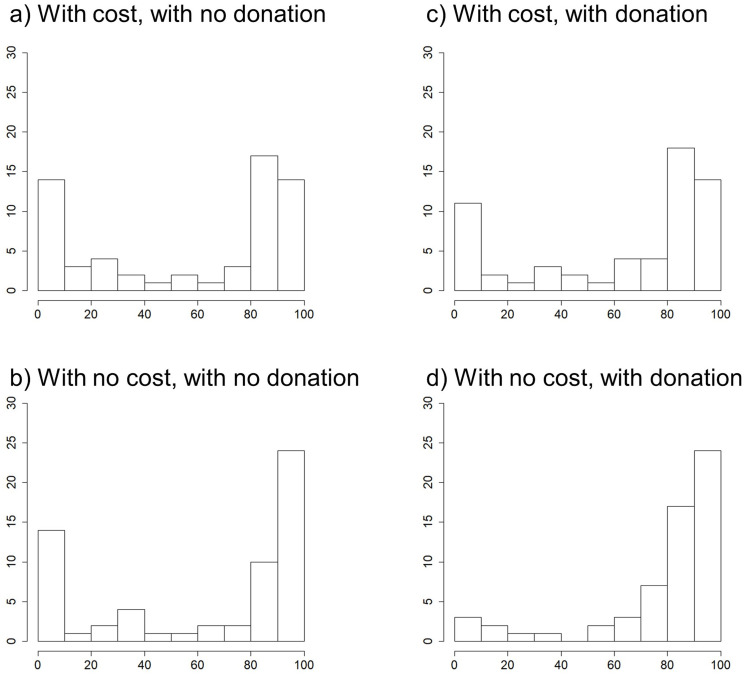
Distributions of correct answers in each treatment. Panel a, b, c and d show treatment A, B, C, D respectively. x axis shows the number of correct answers and y axis shows the number of participants.

We first analyze distributions of the correct answer ratio in each treatment. Fig 2 is a histogram of the distributions of correct answers.

As shown in Fig 2, respondents were answering either almost all of the questions correctly or answering almost none of them correctly. The results suggest that participants behaved as a full cooperator or a full defector in each treatment. The purpose of this paper is to determine how the two incentives affected cooperation and defection. As most participants can be classified as full cooperator or full defector, we analyze the effect of the two incentives on full cooperator / full defector and clarify the impact of the incentives on prosocial behavior. Since the distributions of participants’ contributions were bimodal, we examine the effect of incentives in a multiple regression model, instead of comparing each case directly. Therefore, we add a dummy variable for the number of correct answers above 90 as 1 and the number of correct answers below 90 as 0, denoted as fullC. In addition, we add a dummy variable for the number of correct answers less than or equal to 10 as 1 and greater than 10 as 0, denoted as fullD (see S1 Text).

We performed a binominal logistic regression analysis that used as dependent variables fullC and fullD. The variables in the model include demographic characteristics, namely age, and gender. Tables 1 and 2 report odd ratios (ORs) and 95% confidence intervals from logistic regressions. Table 1 shows the results of model 1, which examines the effects of the incentives.

**Table 1:** Effect of incentives on full cooperation and defection.

	fullC			fullD		
Variable name	ORs		95% CI	ORs		95% CI
gender	.626		0.354-1.106	.785		0.381-1.618
age	.969	**	0.948-0.991	.982		0.952-1.014
economic incentive	1.103		0.639-1.903	.448	*	0.222-0.903
social incentive	2.160	**	1.249-3.737	.616		0.312-1.215
*R*2	.085	**		.076	+	

^+^ *p* < .1.

* *p* < .05.

** *p* < .01.

**Table 2:** Effect of incentives on full cooperation and defection including psychological attitudes.

	fullC			fullD		
Variable name	ORs		95% CI	ORs		95% CI
gender	.647		0.364-1.151	.847		0.399-1.799
age	.967	**	0.946-0.989	.982		0.950-1.014
economic incentive	1.086		0.628-1.877	.494	+	0.241-1.010
social incentive	2.207	**	1.269-3.838	.652		0.324-1.311
generalized trust	1.075		0.930-1.242	.953		0.791-1.148
generalized reciprocity	1.002		0.844-1.190	.815	*	0.671-0.990
*R*2	.091	*		.111	*	

^+^ *p* < .1.

* *p* < .05.

** *p* < .01.

To consider effects of psychological attitudes, generalized trust [29, 30] and reciprocity [31] were introduced into the regression. To measure generalized trust, subjects were asked to rate the following two items: “Most people are trustworthy” and “Most people trust others”. A subject’s score on generalized trust was obtained by simple addition of the two items’ scores (*α* = .819). Two statements on a questionnaire were used to measure the subjects’ level of reciprocity. Their overall score was obtained by a simple addition of the scores of the responses to the two statements. The two items used to calculate reciprocity were: “When someone helps me, I also help someone else”, and “I believe that good things eventually come back to me when I am kind to others” (*α* = .685). Table 2 shows the model 2, which controls the effects of the incentives and examines the effects of psychological attitudes.

In both model 1 and 2, on the one hand, we find that social incentives have a positive effect on fullC while economic incentives do not have a significant effect. On the other hand, for fullD, economic incentives have a significant effect. These results indicate that social incentives promote full cooperation and economic incentives reduce **full defection / non-cooperation**. Also, the age variable is significant for fullC. After controlling for incentives, a higher score on general reciprocity had the effect of reducing non-cooperation. The generalized trust score had no statistically significant effect.

## Discussion

Analyzing factors that induce cooperative behavior in real effort based public goods games is quite important from a practical point of view as to whether or not incentives are efficient in respect to their type and who they are given to, especially when promoting environmentally conscious policies such as the Vienna Kultur-Token. Our results provide objective experimental evidence identifying incentives that promote cooperative behavior and those that inhibit non-cooperative behavior.

In our online experiment involving 242 subjects, we show two statistically significant factors in promoting the number of full cooperators. Participants are classified as full cooperators, if they show extensive cooperation over all 100 image recognition tasks and answer 90 or more correctly. One is the age effect. The older a person, the more fully cooperative. Similar experiments have shown that age matters for social preferences [37–39], and we have added another piece of evidence that this result is robust.

Our finding with statistically strong significance that a social incentive is effective in promoting full cooperators is expected to further inform real policies, despite little academic accumulation. Especially, it might be applied and tested in a concrete challenge of promoting environmentally conscious behavior using the Vienna Kutur-Token. For example, the city of Vienna can communicate to citizens “Our city will make a donation to the Red Cross corresponding to the amount of carbon dioxide reduced as a result of your eco-friendly behavior”. Of course, our experiment is based on an image recognition task, and it is necessary to consider that there is a qualitative difference from the real effort in the Vienna Kultur-Token project.

Our experiments show not only two factors that promote full cooperation, but also two factors that suppress the number of full defectors. Participants are classified as full defectors, if they show a minimum to no cooperation over all 100 image recognition tasks and answer only 10 or fewer correctly. One is that participants with a high score on the generalized reciprocity scale have statistically significantly fewer full defectors than those without. Evolutionary studies have confirmed that combined with certain behavioral strategies, generalized reciprocity could bring the benefits of generating cooperation behaviors and promoting interpersonal communication [40, 41]. Our result supports similar experiments with the generalized reciprocity scale that show the same direction of results [42].

It should be emphasized that the two incentives have asymmetric effects on people’s contributions. Intuitively, economic incentives are supposed to promote cooperation, but our results show that economic incentives have an effect on cooperation in the form of a reduction in full defection. On the other hand, social incentives promote full cooperation even though people do not directly receive the additional benefits from cooperation. We may think that a positive effect resulting from positive incentives can be predictable. However, results from experiments with real effort are not that common and can offer an interesting research avenue allowing to further generalize and then measure quantitatively the effects of real effort. We also believe that the results concerning sensitivity for economic incentives for those that cooperate extensively and social incentives for those that cooperate barely at all are promising and worthy of further discussion.

We must point out that as a limitation of this research, this is an analysis based on a Japanese crowd-working platform and its comparability to other real effort subjects needs to be further researched. In future extensions, international comparisons and an application to other types of real effort tasks should be considered. Additionally, the current form does not allow us to generalize a quantification of the cost of effort which restricts our ability to analyze the setting via the Social Efficiency Deficit [43]. Future research should attempt to generalize such limitations via indices to increase comparability.

In this paper, we conduct an online experiment and lead some results in a framework of a real effort based public goods game that has not received much attention until now. In particular, our finding that social and economic incentives have a certain effect on social order in different ways provides further testing ground in the field study of the Vienna Kultur-Token project, but also future academic lab settings.

## Supporting information

S1 Text (PDF)
