# Supplementary material for: Asymmetric effects of social and economic incentives on cooperation in real effort based public goods games
Source: PLoS One. 2021 Apr 14;16(4):e0249217. doi: 10.1371/journal.pone.0249217 (PMC8046186; doi:10.1371/journal.pone.0249217)
Supplement: S1 Text — (PDF) [file pone.0249217.s001.pdf]

Asymmetric effects of social and economic incentives on cooperation in real effort based public goods games

Jakob Hackel[1*], Hitoshi Yamamoto[1,2], Isamu Okada[1,3], Akira Goto[4], Alfred Taudes[1]

1 Research Institute for Cryptoeconomics, University of Economics and Business, Vienna, Austria
2 Faculty of Business Administration, Rissho University, Tokyo, Japan
3 Faculty of Business Administration, Soka University, Tokyo, Japan
4 School of Information and Communication, Meiji University, Tokyo, Japan

* jakob.hackel@wu.ac.at (JH)

This file includes: Supplementary Text S1 - S3 Supplementary Tables 1 – 2 Supplementary Figures 1 – 9

Text S1

Manipulation check

The following two questions were used to check whether the participants understood the experimental setup. "The number of tasks you complete (correctly labeled) will affect whose rewards?" (1) Only one of the group members (2) Only one of the participants in the experiment. "No" was the correct answer for both items; we included 242 participants in our analysis who answered both questions correctly, although 350 participants completed the experiment.

Text S2

Analysis

To test the validity of the thresholds, we tested the effect of incentives with three patterns of fullC thresholds of 90, 85, and 80. Similarly, we validated the thresholds for fullD with three patterns of 10, 15, and 20. Overall, similar trends were observed when the threshold was changed in fullC and fullD, although the effect of the social incentive disappeared when the threshold was set at 80% for fullC. Therefore, we employed the thresholds of 90 and 10 for our paper to clearly analyze the factors of "full" cooperation and defection.

Text S3

Experiment Instructions

Experiment Instructions for treatment A (translated)

Here you will participate in a simple game. You will receive an additional payment (Note by author: additional to 5 JPY as show-up fee, which participants were informed of before) for Gold that you gain during the game. The conversion rate for your additional Gold is [1 Gold =0.1 JPY]. Your earnings will be paid out over the next couple of days.

Variable name	C90		C85		C80
q_gender	.647		.770		.857
q_age	.967	**	.978	+	.991
e_incentive	1.086		1.047		1.343
s_incentive	2.207	**	1.852	*	1.400
trust	1.075		1.067		1.046
recipro	1.002		.997		1.050
R^2	.091	*	.050		0.25

$^+p < .1$. $^*p < .05$. $^{**}p < .01$.

Table 1. Variable fullC thresholds

Variable name	D10		D15		D20	
q_gender	.847		1.113		1.146	
q_age	.982		.983		.987	
e_incentive	.494	+	.534	+	.538	+
s_incentive	.652		.684		.622	
trust	.953		.935		.952	
recipro	.815	*	.856	+	.870	
R^2	.111	*	.092	+	0.83	+

$^+p < .1$. $^*p < .05$. $^{**}p < .01$.

Table 2. Variable fullD thresholds

- This game is played as a party of five people. A party is determined randomly.
- You will be given 100 initial Gold
- You will be shown 100 tasks on image-recognition.
- For every task that you solve correctly, **everyone else in your group will receive 1 Gold each.**
- **Any task you want to start costs 1 Gold each.**
- For every task anyone else in your group solves correctly, you will receive 1 Gold.
- Please note that any task you solve correctly will only award points to the other members of your group and you lose 1 Gold.
- The other members of the group will never be revealed. You will not know who they are, and they will not know who you are.

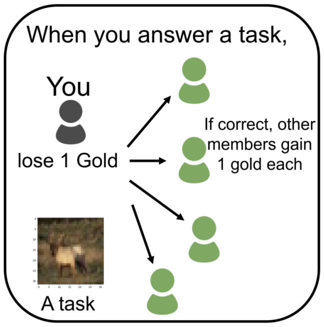

Fig 1. Punch images for treatment A

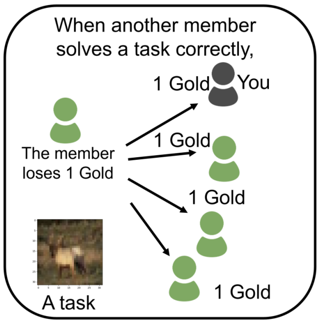

Fig 2. Punch images for treatment A

- **You can decide to stop working on tasks at any time. There are no penalties for stopping early. All your correctly solved tasks will still count for the rest of your group.**
- **Other people solving these tasks, including your group, will never be informed about the number of tasks you solved or when you stopped.**
- **At the end you will receive Gold. This is a total of all Gold you received. You will not know who was in your group or how many tasks anyone else solved. Similarly, no one will receive information about the number of tasks you solved.**

Are you ready? If you understand the explanation above, go to the next page to start your tasks.

Experiment Instructions for treatment B (translated)

Here you will participate in a simple game. You will receive an additional payment (Note by author: additional to 5 JPY as show-up fee, which participants were informed of before) for Gold that you gain during the game. The conversion rate for your additional Gold is [1 Gold =0.1 JPY]. Your earnings will be paid out over the next couple of days.

- This game is played as a party of five people. A party is determined randomly.
- You will be given 100 initial Gold
- You will be shown 100 tasks on image-recognition.
- For every task that you solve correctly, **everyone else in your group will receive 1 Gold each. Additionally, for every task you solve correctly, we will donate 1JPY to the Red Cross.**
- **Any task you want to start costs 1 Gold each.**
- For every task anyone else in your group solves correctly, you will receive 1 Gold.
- Please note that any task you solve correctly will only award points to the other members of your group and you lose 1 Gold.
- The other members of the group will never be revealed. You will not know who they are, and they will not know who you are.

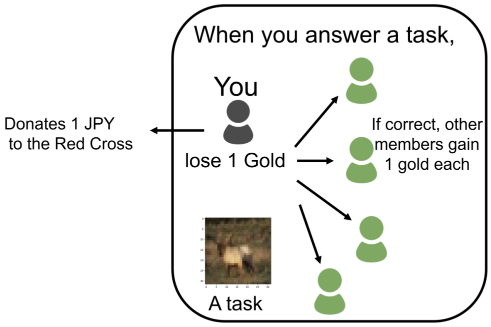

Fig 3. Punch images for treatment B

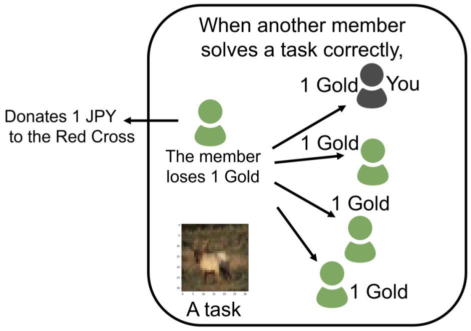

Fig 4. Punch images for treatment B

- **You can decide to stop working on tasks at any time. There are no penalties for stopping early. All your correctly solved tasks will still count for the rest of your group.**
- **Other people solving these tasks, including your group, will never be informed about the number of tasks you solved or when you stopped.**
- **At the end you will receive Gold. This is a total of all Gold you received. You will not know who was in your group or how many tasks anyone else solved. Similarly, no one will receive information about the number of tasks you solved.**

Are you ready? If you understand the explanation above, go to the next page to start your tasks.

Experiment Instructions for treatment C (translated)

Here you will participate in a simple game. You will receive an additional payment (Note by author: additional to 5 JPY as show-up fee, which participants were informed of before) for Gold that you gain during the game. The conversion rate for your additional Gold is [1 Gold =0.1 JPY]. Your earnings will be paid out over the next couple of days.

- This game is played as a party of five people. A party is determined randomly.
- You will be given 100 initial Gold
- You will be shown 100 tasks on image-recognition.
- For every task that you solve correctly, **all members including you in your group will receive 1 Gold each.**
- **Any task you want to start costs 1 Gold each.**
- For every task anyone else in your group solves correctly, you will receive 1 Gold.
- The other members of the group will never be revealed. You will not know who they are, and they will not know who you are.

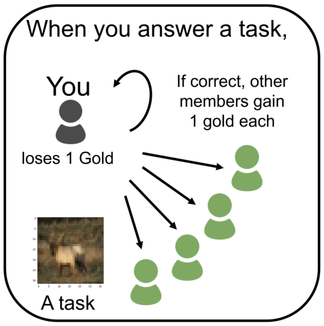

Fig 5. Punch images for treatment C

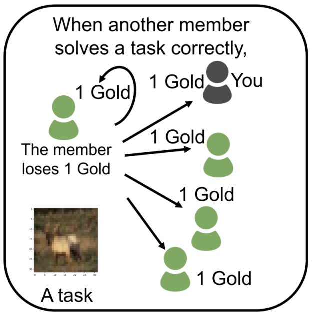

Fig 6. Punch images for treatment C

- **You can decide to stop working on tasks at any time. There are no penalties for stopping early. All your correctly solved tasks will still count for the rest of your group.**

- **Other people solving these tasks, including your group, will never be informed about the number of tasks you solved or when you stopped.**
- **At the end you will receive Gold. This is a total of all Gold you received. You will not know who was in your group or how many tasks anyone else solved. Similarly, no one will receive information about the number of tasks you solved.**

Are you ready? If you understand the explanation above, go to the next page to start your tasks.

Experiment Instructions for treatment D (translated)

Here you will participate in a simple game. You will receive an additional payment (Note by author: additional to 5 JPY as show-up fee, which participants were informed of before) for Gold that you gain during the game. The conversion rate for your additional Gold is [1 Gold =0.1 JPY]. Your earnings will be paid out over the next couple of days.

- This game is played as a party of five people. A party is determined randomly.
- You will be given 100 initial Gold
- You will be shown 100 tasks on image-recognition.
- For every task that you solve correctly, **all members including you in your group will receive 1 Gold each. Additionally, for every task you solve correctly, we will donate 1JPY to the Red Cross.**
- **Any task you want to start costs 1 Gold each.**
- For every task anyone else in your group solves correctly, you will receive 1 Gold.
- The other members of the group will never be revealed. You will not know who they are, and they will not know who you are.

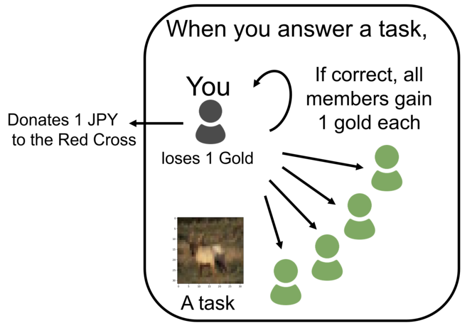

Fig 7. Punch images for treatment D

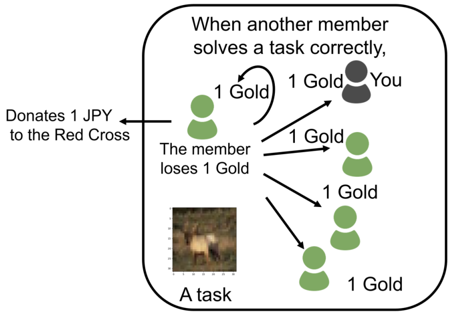

Fig 8. Punch images for treatment D

- **You can decide to stop working on tasks at any time. There are no penalties for stopping early. All your correctly solved tasks will still count for the rest of your group.**
- **Other people solving these tasks, including your group, will never be informed about the number of tasks you solved or when you stopped.**

- **At the end you will receive Gold. This is a total of all Gold you received. You will not know who was in your group or how many tasks anyone else solved. Similarly, no one will receive information about the number of tasks you solved.**

Are you ready? If you understand the explanation above, go to the next page to start your tasks.

Tasks on image recognition

Participants are shown 100 tasks on image recognition in a screen with a scroll. All images come from Cifar-10 dataset. Fig. S5 is a sample image. In each task, participants choose one among 11 options consisting of 10 categories and [do not answer], where the 10 categories are airplane, automobile, bird, cat, deer, dog, frog, horse, ship, and truck, respectively. In the top of this screen, the following sentences are shown: If you would like to stop solving tasks and earning Gold for your group, you can stop at any time. There is no penalty and the tasks you already solved will count in full. If you click the QUIT button below, no more tasks will be shown, and you will skip forward to the questionnaire.

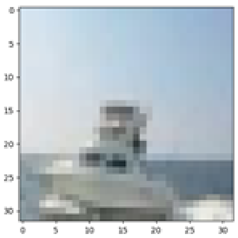

Fig 9. SI5 A sample picture Original from CIFAR-10 dataset

Questionnaire items (translated)

After performing tasks on image recognition, participants go to pages on questionnaires including demographic traits, psychological traits, and a manipulation check. The psychological traits we take as the questionnaire here are 1) trust and 2) reciprocity. All questionnaire items and their options are shown in SI-dataset.
